# Breed- and age-associated variation in hematological parameters and Toll-like receptor 2 gene expression in peripheral blood mononuclear cells of commercial and purebred laying hens during the laying cycle

**DOI:** 10.14202/vetworld.2026.3558-3567

**Published:** 2026-08-11

**Authors:** Tiprawee Rassamepong, Panwadee Sopannarath, Muhammad Jasim Uddin, Anchalee Buadkhunthod, Niparat Khotanon, Autchara Kayan

**Affiliations:** 1 Department of Animal Science, Faculty of Agriculture, Kasetsart University, Bangkok 10900, Thailand.; 2 School of Veterinary Medicine, Murdoch University, Western Australia 6155, Australia.

**Keywords:** gene expression, hematological parameters, innate immunity, laying hens, peripheral blood mononuclear cells, physiological stage, Toll-like receptor 2, White Leghorn

## Abstract

**Background and Aim:**

Hematological parameters and innate immune responses are influenced by genetic background and physiological status, making them valuable indicators of health and immune competence in laying hens. However, comparative information on breed- and age-related variation in hematological characteristics and *Toll-like receptor* *2* (*TLR2*) gene expression in PBMCs during the laying cycle remains limited. This study evaluated the effects of breed and age on hematological traits and *TLR2* gene expression in commercial Lohmann Brown (LB) and purebred White Leghorn (WL) laying hens to improve the understanding of innate immune regulation during different stages of egg production.

**Materials and Methods:**

Twenty clinically healthy laying hens (n = 5 per breed × age group) comprising LB and WL breeds at 42 and 80 weeks of age were maintained under identical management conditions. Blood samples were collected for hematological analysis and PBMC isolation. Hematological parameters were determined using standard laboratory methods, whereas *TLR2* gene expression was quantified by real-time quantitative polymerase chain reaction. Data were analyzed using a completely randomized 2 × 2 factorial design to evaluate the effects of breed, age, and their interaction.

**Results:**

Breed significantly affected hemoglobin concentration, lymphocyte percentage, heterophil-to-lymphocyte (H:L) ratio, and *TLR2* expression (p < 0.05). WL hens exhibited higher hemoglobin concentration, H:L ratio, and *TLR2* expression than LB hens, whereas LB hens had higher lymphocyte percentages. Age significantly influenced hematocrit, hemoglobin, heterophil, lymphocyte, monocyte, white blood cell count, H:L ratio, and *TLR2* expression (p < 0.05). Hens at 80 weeks exhibited higher hematocrit, hemoglobin, lymphocyte percentage, monocyte percentage, and white blood cell count, whereas hens at 42 weeks had higher heterophil percentage, H:L ratio, and *TLR2* expression. Significant breed × age interactions were observed for hemoglobin, heterophil, lymphocyte, H:L ratio, and *TLR2* expression, with WL hens at 42 weeks exhibiting the highest *TLR2* expression. *TLR2* expression showed positive correlations with the heterophil percentage and the H:L ratio, but a negative correlation with the lymphocyte percentage.

**Conclusion:**

Breed and physiological stage significantly influence hematological characteristics and *TLR2*-mediated innate immune responses in laying hens. The combined evaluation of hematological parameters and *TLR2* expression provide complementary biomarkers for assessing immune competence and physiological stress during the laying cycle. These findings establish baseline reference data that may facilitate genetic selection for disease resilience, improve flock health monitoring, and support precision breeding and management strategies for sustainable egg production.

## INTRODUCTION

Laying hens play a crucial role in the global food industry, and understanding the factors influencing their health and productivity is essential for improving layer management [1]. Lohmann Brown (LB) and White Leghorn (WL) are among the most widely used laying hen breeds worldwide. These breeds differ considerably in genetic background, selection history, productivity, and physiological characteristics. Commercial strains such as LB have undergone intensive selection for egg production traits, whereas WL represents a more traditional genetic line commonly used as a reference breed in poultry research. These genetic differences may influence immune regulation and hematological characteristics. However, comparative information regarding innate immune-related gene expression, particularly *Toll-like receptor* *2* (*TLR2*), between these breeds remains limited. Breed- and age-related changes can also affect immune characteristics in chickens. Immune activity generally increases during the early growth stage but tends to decline during the laying period, with a shift toward humoral and adaptive immune responses. Such changes may influence the health status, immune competence, and productivity of laying hens [2].

Furthermore, pathogen infections can significantly affect the immune system and remain a major challenge in poultry production. In Thailand, bacterial pathogens such as *Salmonella*, *Escherichia coli*, and *Clostridium* spp. are commonly reported. These pathogens can negatively affect animal health, growth performance, productivity, and product quality [3]. Hematological parameters are widely used as indicators of physiological status and health in poultry [4]. Toll-like receptors (TLRs) are essential components of the innate immune system. They function as pattern-recognition receptors that detect pathogen-associated molecular patterns (PAMPs) from microbial pathogens [5]. These membrane-bound receptors recognize both external pathogens and endogenous danger signals. They initiate immune responses to maintain host defense. Activation of TLR signaling pathways stimulates both innate and adaptive immune responses, leading to the production of cytokines and other immuno-modulatory molecules [6].

*TLR2* was selected as the target gene because it is one of the major pattern-recognition receptors involved in the detection of pathogen-associated molecular patterns (PAMPs) derived from Gram-positive and Gram-negative bacteria, fungi, viruses, and parasites. Activation of *TLR2* initiates innate immune signaling pathways and promotes the production of inflammatory mediators and cytokines that contribute to host defense mechanisms [5–8]. Therefore, changes in *TLR2* expression may reflect alterations in immune responsiveness associated with age and genetic background in laying hens. In commercial layer production, approximately 42 weeks of age represents the peak-laying period with maximum egg production and high metabolic and physiological demands. In contrast, 80 weeks of age represents the late-laying period, characterized by age-related physiological changes and alterations in immune function. Comparing these two production stages may provide valuable insights into age-associated changes in hematological characteristics and innate immune responses.

Although age-related changes in hematological and immune parameters of laying hens have been reported, previous studies have primarily focused on blood profiles, production performance, or general immune responses [2, 4]. In contrast, information regarding the expression of innate immune recognition genes, particularly *TLR2*, in peripheral blood mononuclear cells (PBMCs) during different physiological stages of the laying cycle remains scarce. PBMCs play a central role in immune surveillance and host defense. Because *TLR2* is expressed in immune cells and functions as a key pattern-recognition receptor, its expression in PBMCs may serve as a useful indicator of innate immune activation and responsiveness during different physiological stages of the laying cycle. Previous studies have mainly investigated *TLR2* expression in response to pathogen challenge or among different chicken breeds [7, 8]. However, comparative information on *TLR2* expression between peak-laying and late-laying hens, particularly across distinct genetic lines such as LB and WL, remains limited. Hematological parameters provide information on the physiological and health status of birds, whereas *TLR2* expression reflects innate immune recognition mechanisms at the molecular level. Evaluating both hematological traits and *TLR2* gene expression may provide a more comprehensive understanding of age- and breed-related differences in immune regulation than either approach alone.

Therefore, the objective of this study was to evaluate the effects of breed (LB vs. WL) and age (peak-laying at 42 weeks vs. late-laying at 80 weeks) on hematological parameters and *TLR2* gene expression in PBMCs of laying hens during the laying period. This research is expected to provide insights into breed- and age-specific immune profiles. These findings may support improved management strategies for maintaining health and productivity in commercial laying hen flocks.

## MATERIALS AND METHODS

### Ethical approval

The study protocol was reviewed and approved by the Animal Ethics Committee of Kasetsart University, Bangkok, Thailand (Approval No. ACKU66-AGR-018). All procedures involving laying hens were conducted in accordance with the approved institutional protocol and applicable guidelines for the ethical care and use of animals in research. The study was designed to minimize animal use and discomfort while obtaining scientifically valid data, consistent with the principles of replacement, reduction, and refinement.

Only clinically healthy LB and WL laying hens were included. Before enrolment, all birds were visually examined and confirmed to be free from apparent clinical signs of disease. The hens were maintained under uniform housing, feeding, lighting, ventilation, vaccination, and management conditions at the Animal Research Farm, Department of Animal Science, Kasetsart University. Animal health and welfare were monitored throughout the study, and blood sampling was performed by trained personnel using appropriate handling and restraint procedures to minimize stress and discomfort.

A total of 2 mL of blood was collected once from the brachial vein of each hen into an ethylenediaminetetra-acetic acid-coated tube for hematological analysis and peripheral blood mononuclear cell isolation. The sampling volume was limited to that required for the planned analyses, and no experimental infection, surgical intervention, prolonged restraint, or terminal procedure was performed. Following blood collection, the birds remained under routine farm management and observation. The sample size was limited to the minimum number of animals deemed necessary, based on animal availability, ethical considerations, and previous poultry studies evaluating hematological and immune-related gene expression parameters.

### Study period and location

The experiment was conducted from September 2023 to September 2025 at the Animal Research Farm, Department of Animal Science, Kasetsart University, Thailand.

### Study design

A total of 20 clinically healthy laying hens were selected from LB (population size = 1,381) and WL (population size = 79) flocks at 42 and 80 weeks of age. All birds were visually examined and confirmed to be free from clinical signs of disease. Five hens were then randomly chosen from each breed and age group under the same housing and management conditions for blood collection and subsequent analyses. All hens were housed in conventional layer cages under the same environmental and management conditions. The house was equipped with mechanical ventilation and maintained in accordance with standard commercial management practices. A 16-h light program was provided throughout the study. Birds were fed a commercial diet (120 g/day; 17% crude protein and 3,500 kcal ME/kg) with *ad libitum* access to water. All birds were clinically healthy at the time of sampling and showed no apparent signs of disease. The flock was managed according to the routine vaccination program of the research farm. A formal *a priori* statistical power analysis was not performed. The sample size was determined based on ethical considerations, animal availability, and previous poultry studies evaluating hematological and immune-related gene expression parameters.

### Sample collection

Blood samples (2 mL) were randomly collected from the brachial vein of laying hens (n = 5 per breed and age) into ethylenediaminetetraacetic acid-coated tubes to prevent coagulation. Each sample was divided into two aliquots: 1 mL for hematological analysis and 1 mL for PBMC isolation. Blood samples were transported to the laboratory immediately after collection and processed on the same day. Hematological analyses were performed within a few hours of collection to minimize storage-related alterations in blood cell characteristics. PBMC isolation was performed immediately after blood collection.

### Hematological analysis

Hematological parameters, including red blood cells (RBC), hemoglobin, hematocrit, white blood cells (WBC), lymphocytes, monocytes, basophils, eosinophils, and heterophils, were determined using a Neubauer hemocytometer following dilution (1:200) with Natt and Herrick solution, according to the method described by [9].

### PBMC isolation

PBMCs were chosen as they represent a readily accessible, non-invasive source of immune cells that reflect systemic innate immune status. PBMCs were isolated from whole blood following the method of [10]. Briefly, 1 mL of blood was layered over 2 mL of Lymphoprep™ (Stemcell Technologies, Cologne, Germany) on first use and centrifuged at 277 × *g* for 30 min. The mononuclear cell layer was collected and stored at −80 °C for further analysis.

### RNA isolation and gene expression analysis

Total RNA was extracted from PBMCs using a QIAamp RNA Mini Kit (Qiagen, Courtaboeuf, France) on first use according to the manufacturer’s instructions. RNA purity was assessed using a NanoDrop spectrophotometer (A260/280 ratio of 1.80–2.10). Gene expression analysis was performed using a MyGo Pro® real-time polymerase chain reaction (PCR) system (IT-IS Life Science Ltd., Middlesbrough, UK) on first use with the QuantiNova SYBR Green RT-PCR Kit (Qiagen, Hilden, Germany) on first use. This one-step RT-qPCR system combines reverse transcription and quantitative PCR amplification in a single reaction. Reactions were prepared according to the manufacturer’s instructions, and amplification was performed on a real-time PCR instrument under the recommended cycling conditions. Each 20 µL reaction contained 10 µL of 2× master mix, 1 µL each of forward and reverse primers (10 µM), 0.2 µL of RT mix, 5 µL RNA template, and nuclease-free water. Amplification conditions consisted of pre-denaturation at 95 °C for 2 min followed by 40 cycles of 95 °C for 5 s, 60 °C for 10 s, and 72 °C for 15 s. Melting curve analysis was performed from 60 to 97 °C. Each sample was analyzed in duplicate, and gene expression levels were calculated from the quantification cycle ratio of the target gene to the housekeeping gene *β-actin*, which is widely used as a stable reference gene in avian gene expression studies [11]. Primer sequences are shown in Table 1.

**Table 1:** qRT-PCR primer sequences.

**Gene**	**Primer sequence**	**Tm (°C)**	**Reference**
**β-actin**	FW: 5′-CCACCGCAAATGCTTCTA-3′ RW: 5′-GCCAATCTCGTCTTGTTTTATG-3′	60	[11]
**TLR2**	FW: 5′-CTGATCCTGTGCCAATCAGA-3′ RW: 5′-CCTGGTGCTCCATCTCAAGT-3′	60	[11]

FW = Forward primer; qRT-PCR = Quantitative real-time polymerase chain reaction; RW = Reverse primer; TLR2 = Toll-like receptor 2; Tm = Melting temperature.

### Statistical analysis

Data were analyzed using the General Linear Model (GLM) procedure of SAS statistical software version 9.2 (SAS Institute Inc., Cary, NC, USA) in a completely randomized 2 × 2 factorial arrangement, with breed (LB and WL) and age (42 and 80 weeks) as the main factors. Prior to analysis, data were evaluated for normality and homogeneity of variance. When significant effects were detected, least squares means were compared using Tukey’s multiple comparison test. Differences were considered significant at p < 0.05. The results were presented as least squares means with standard errors. The model used in statistics was:

Y_ijk_ = µ + α_i_ + β_j_ + (αβ)_ij_ + ε_ijk_

where Y_ijk_ = the value of individual samples of the data from each replicate, µ = overall mean, α_i_ = breed effect (i = LB and WL), β_j_ = age effect (j = 42 and 80 weeks of age), (αβ)_ij_ = interaction between breed and age, and ε_ijk_ = the random residual error.

## RESULTS

### Hematological parameters

The hematological parameters were affected by laying hen breeds, including hemoglobin, lymphocyte count, and the heterophil-to-lymphocyte (H:L) ratio (Table 2). The results showed that the hemoglobin concentration in WL hens was significantly higher than in LB hens (15.32 ± 0.55 vs. 12.42 ± 0.59 g/dL) (p < 0.01). LB hens had a significantly higher lymphocyte level compared to WL hens (62.70 ± 2.54 vs. 53.90 ± 2.39%, respectively) (p < 0.05). Additionally, the H:L ratio in WL hens was significantly higher than in LB hens (1.07 ± 0.11 vs. 0.57 ± 0.12, respectively) (p < 0.01). However, there were no significant differences between breeds in basophil, eosinophil, hematocrit, heterophil, monocyte, RBC, and WBC counts (p > 0.05).

The influence of age on hematological parameters was determined. The results revealed that laying hen age affected hematocrit, hemoglobin, heterophil, lymphocyte, H:L ratio, monocyte, and WBC counts (p < 0.05) (Table 2). Laying hens at 80 weeks of age had a significantly higher hematocrit level compared to laying hens at 42 weeks of age (33.00 ± 1.11 vs. 27.40 ± 1.18%, respectively) (p < 0.01). Meanwhile, the hemoglobin concentration in laying hens at 80 weeks of age was significantly higher than in laying hens at 42 weeks of age (16.52 ± 0.55 vs. 11.22 ± 0.59 g/dL) (p < 0.001). The lymphocyte, monocyte, and WBC levels in laying hens at 80 weeks of age were 68.60 ± 2.39%, 2.10 ± 0.29%, and 10.88 ± 0.83 × 10^3^ cells/mm^3^, respectively. These values were 48.00 ± 2.54%, 1.10 ± 0.31%, and 6.68 ± 0.88 × 10^3^ cells/mm^3^, respectively, in laying hens at 42 weeks of age. However, the heterophil level in laying hens at 42 weeks of age was significantly higher compared to laying hens at 80 weeks of age (48.98 ± 2.90 vs. 27.60 ± 2.73%) (p < 0.001). In addition, the H:L ratio in laying hens at 42 weeks of age was 1.23 ± 0.12, which was significantly higher than in laying hens at 80 weeks of age (0.42 ± 0.11) (p < 0.001). There were no significant differences between ages in basophil, eosinophil, and RBC counts (p > 0.05).

**Table 2:** Hematological parameters of commercial and purebred laying hens at different ages.

**Parameter**	**LB**	**WL**	**42 weeks**	**80 weeks**	**Breed p-value**	**Age p-value**
Basophil (%)	0.78 ± 0.25	0.40 ± 0.24	0.38 ± 0.25	0.80 ± 0.24	0.293	0.236
Eosinophil (%)	0.90 ± 0.44	1.30 ± 0.42	1.30 ± 0.44	0.90 ± 0.42	0.520	0.520
Hematocrit (%)	30.20 ± 1.18	30.20 ± 1.11	27.40 ± 1.18ᵈ	33.00 ± 1.11ᶜ	1.000	0.004
Hemoglobin (g/dL)	12.42 ± 0.59ᴰ	15.32 ± 0.55ᶜ	11.22 ± 0.59ᶠ	16.52 ± 0.55ᵉ	0.003	<0.001
Heterophil (%)	34.18 ± 2.90	42.40 ± 2.73	48.98 ± 2.90ᵉ	27.60 ± 2.73ᶠ	0.057	<0.001
Lymphocyte (%)	62.70 ± 2.54ᴬ	53.90 ± 2.39ᴮ	48.00 ± 2.54ᶠ	68.60 ± 2.39ᵉ	0.024	<0.001
H:L ratio	0.57 ± 0.12ᴰ	1.07 ± 0.11ᶜ	1.23 ± 0.12ᵉ	0.42 ± 0.11ᶠ	0.008	<0.001
Monocyte (%)	1.20 ± 0.31	2.00 ± 0.29	1.10 ± 0.31ᵇ	2.10 ± 0.29ᵃ	0.079	0.033
RBC (×10⁶ cells/mm³)	2.50 ± 0.09	2.39 ± 0.09	2.32 ± 0.09	2.57 ± 0.09	0.386	0.064
WBC (×10³ cells/mm³)	9.86 ± 0.88	7.70 ± 0.83	6.68 ± 0.88ᵈ	10.88 ± 0.83ᶜ	0.094	0.003

H:L ratio = Heterophil-to-lymphocyte ratio; LB = Lohmann Brown; RBC = Red blood cell; WBC = White blood cell; WL = White Leghorn.

ᴬ–ᴮ Means within the same row with different superscripts differ significantly between breeds at p < 0.05. ^C^–ᴰ Means within the same row with different superscripts differ significantly between breeds at p < 0.01. ᵃ–ᵇ Means within the same row with different superscripts differ significantly between ages at p < 0.05. ᶜ–ᵈ Means within the same row with different superscripts differ significantly between ages at p < 0.01. ᵉ–ᶠ Means within the same row with different superscripts differ significantly between ages at p < 0.001.

The interactions between breed and age on hematological parameters are presented in Table 3. There were no significant differences in basophil, eosinophil, hematocrit, monocyte, RBC, and WBC counts (p > 0.05). Significant differences were observed in hemoglobin, heterophil, lymphocyte, and the H:L ratio. The hemoglobin concentrations were the lowest in the LB × 42 group (7.68 ± 0.87 g/dL) (p < 0.001). The heterophil level was the highest in the WL × 42 group (61.20 ± 3.87%) (p < 0.001). The lymphocyte level in the WL × 42 group was the lowest (36.00 ± 3.39%) (p < 0.001). The H:L ratio was the highest in the WL × 42 group (1.81 ± 0.16) (p < 0.001).

**Table 3:** Interaction effects of breed and age on hematological parameters in commercial and purebred laying hens.

**Parameter**	**LB × 42 weeks**	**LB × 80 weeks**	**WL × 42 weeks**	**WL × 80 weeks**	**p-value**
Basophil (%)	0.75 ± 0.37	0.80 ± 0.33	0.00 ± 0.33	0.80 ± 0.33	0.293
Eosinophil (%)	1.00 ± 0.66	0.80 ± 0.59	1.60 ± 0.59	1.00 ± 0.59	0.746
Hematocrit (%)	28.00 ± 1.76	32.40 ± 1.57	26.80 ± 1.57	33.60 ± 1.57	0.470
Hemoglobin (g/dL)	7.68 ± 0.87ᵇ	17.16 ± 0.78ᵃ	14.76 ± 0.78ᵃ	15.88 ± 0.78ᵃ	<0.001
Heterophil (%)	36.75 ± 4.32ᵇ	31.60 ± 3.87ᵇ	61.20 ± 3.87ᵃ	23.60 ± 3.87ᵇ	0.001
Lymphocyte (%)	60.00 ± 3.79ᵃ	65.40 ± 3.39ᵃ	36.00 ± 3.39ᵇ	71.80 ± 3.39ᵃ	<0.001
H:L ratio	0.64 ± 0.18ᵇ	0.50 ± 0.16ᵇ	1.81 ± 0.16ᵃ	0.34 ± 0.16ᵇ	0.001
Monocyte (%)	1.00 ± 0.46	1.40 ± 0.41	1.20 ± 0.41	2.80 ± 0.41	0.179
RBC (×10⁶ cells/mm³)	2.34 ± 0.14	2.66 ± 0.13	2.29 ± 0.13	2.49 ± 0.13	0.669
WBC (×10³ cells/mm³)	7.54 ± 1.31	12.19 ± 1.17	5.83 ± 1.17	9.57 ± 1.17	0.711

H:L ratio = Heterophil-to-lymphocyte ratio; LB = Lohmann Brown; RBC = Red blood cell; WBC = White blood cell; WL = White Leghorn.

ᵃ–ᵇ Means within the same row with different superscripts differ significantly at p < 0.001.

### *TLR2 *gene expression

**Figure 1:**
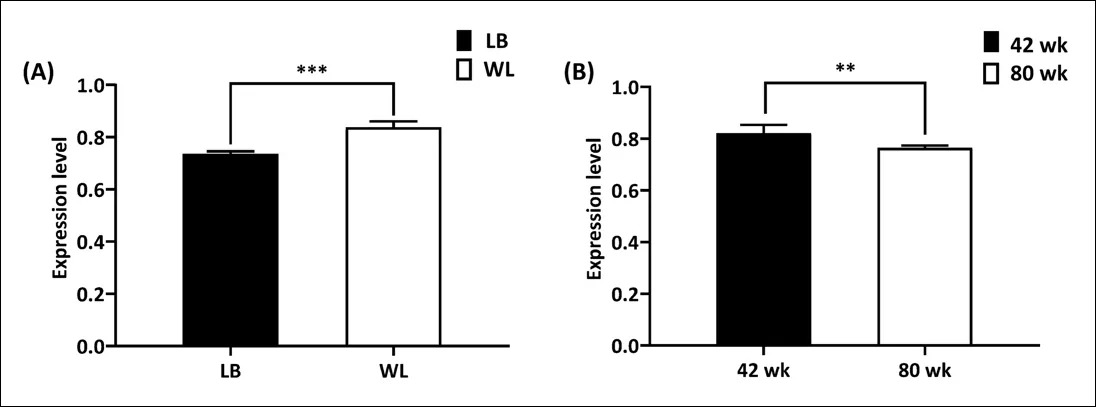
Relative *TLR2* gene expression in laying hens according to breed and age. (A) Comparison between breeds and (B) comparison between ages. Data are presented as mean ± standard error. LB = Lohmann Brown; *TLR2* = Toll-like receptor 2; WL = White Leghorn; wk = Weeks. ⁎⁎ p < 0.01; ⁎⁎⁎ p < 0.001.

*TLR2* gene expression showed significant differences between breeds (p < 0.001) and age (p < 0.01). WL hens had a higher expression level of the *TLR2* gene than LB hens (0.84 ± 0.01 vs. 0.74 ± 0.01, respectively) (Figure 1). 

**Figure 2:**
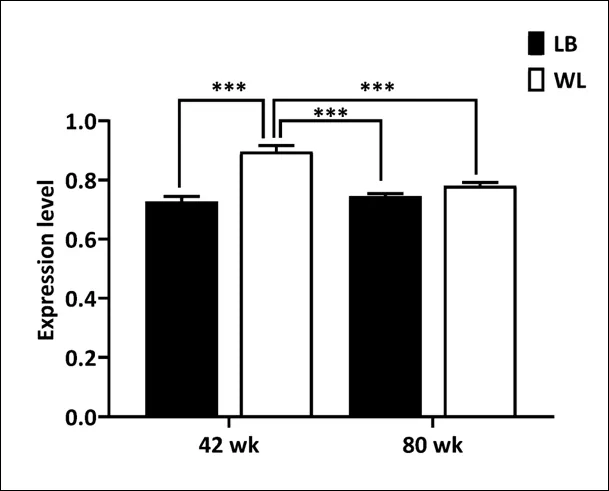
Relative *TLR2* gene expression showing the interaction between breed and age in laying hens. Data are presented as mean ± standard error. LB = Lohmann Brown; *TLR2* = Toll-like receptor 2; WL = White Leghorn; wk = Weeks. ⁎⁎⁎ p < 0.001.

The expression of the *TLR2* gene in laying hens at 42 weeks of age was higher than in laying hens at 80 weeks of age (0.82 ± 0.01 vs. 0.76 ± 0.01, respectively) (Figure 1). The interaction between breed and age was significant for *TLR2* gene expression (p < 0.001) (Figure 2). Notably, the highest *TLR2* expression was observed in WL hens at 42 weeks (0.90 ± 0.02). This represents a novel breed × age interaction, suggesting heightened innate immune vigilance during peak production in this genetic line. The expression of the *TLR2* gene in the WL × 80, LB × 42, and LB × 80 groups was 0.78 ± 0.02, 0.73 ± 0.02, and 0.74 ± 0.02, respectively. Pearson correlation analysis demonstrated a significant positive association between the H:L ratio and *TLR2* gene expression (r = 0.784, p < 0.001) (Table 4). The pronounced elevation of the H:L ratio combined with high *TLR2* expression in the WL × 42 group provides original evidence that genetic background modulates the relationship between production-related stress and innate immune activation.

**Table 4:** Correlations between hematological parameters and TLR2 gene expression in laying hens.

**Parameter**	***TLR2*** ** gene**	**Basophil**	**Eosinophil**	**Hematocrit**	**Hemoglobin**	**Heterophil**	**Lymphocyte**	**H:L ratio**	**Monocyte**	**RBC**	**WBC**
*TLR2* gene	–										
Basophil	−0.599⁎	–									
Eosinophil	−0.020	0.224	–								
Hematocrit	−0.389	0.406	−0.027	–							
Hemoglobin	0.164	0.023	0.088	0.608⁎⁎	–						
Heterophil	0.739⁎⁎⁎	−0.552⁎	−0.085	−0.718⁎⁎⁎	−0.180	–					
Lymphocyte	−0.739⁎⁎⁎	0.518⁎	0.000	0.711⁎⁎⁎	0.167	−0.994⁎⁎⁎	–				
H:L ratio	0.784⁎⁎⁎	−0.481⁎	−0.0662	−0.590⁎⁎	−0.065	0.962⁎⁎⁎	−0.963⁎⁎⁎	–			
Monocyte	−0.221	0.198	−0.010	0.378	0.360	−0.393	0.341	−0.319	–		
RBC	−0.441	0.165	0.2333	0.772⁎⁎⁎	0.563⁎	−0.498⁎	0.482⁎	−0.427	0.194	–	
WBC	−0.557⁎	0.257	0.067	0.547⁎	0.454	−0.584⁎⁎	0.584⁎⁎	−0.584⁎⁎	0.297	0.571⁎	–

H:L ratio = Heterophil-to-lymphocyte ratio; RBC = Red blood cell; *TLR2* = Toll-like receptor 2; WBC = White blood cell. ⁎ p < 0.05; ⁎⁎ p < 0.01; ⁎⁎⁎ p < 0.001.

## DISCUSSION

### Hematological parameters

This study provides original comparative data demonstrating that both breed and physiological stage significantly modulate hematological traits and *TLR2*-mediated innate immunity in laying hens, revealing patterns not previously reported in the literature. Both chicken breed and age significantly influenced hematological parameters in laying hens, reflecting differences in physiological status and immune regulation during the laying cycle. Breed significantly affected hemoglobin concentration, lymphocyte levels, and the heterophil-to-lymphocyte (H:L) ratio, whereas age influenced hematocrit, hemoglobin, heterophil, lymphocyte, monocyte, and total WBC counts. In addition, significant breed–age interactions were observed for hemoglobin, heterophils, lymphocytes, and the H:L ratio. Hemoglobin plays a key role in oxygen transport and reflects the oxygen-carrying capacity of blood [12], and lower hemoglobin concentrations may indicate reduced erythropoiesis or changes in metabolic demand [13]. In the present study, hens at 42 weeks of age exhibited lower hemoglobin levels than those at 80 weeks of age, consistent with previous findings indicating that hemoglobin levels decrease during the peak-laying period and increase again during later stages of production [4]. However, all hematological values remained within normal physiological ranges for healthy laying hens [4, 14], indicating that the birds were not anemic [15]. Age-related changes in hemoglobin levels may reflect adjustments in erythropoiesis associated with reproductive activity and metabolic demand during egg production [16, 17].

Differences in leukocyte profiles further indicated variations in immune responses between breeds and ages. LB hens showed higher lymphocyte levels than WL hens, suggesting breed-related differences in immune regulation and adaptability [13, 18]. In contrast, heterophil levels and the H:L ratio were higher in hens at 42 weeks than at 80 weeks, indicating increased physiological stress during the peak-laying period. Heterophils are primary phagocytic cells involved in innate immune defense against pathogens [19] and increases in heterophil counts accompanied by decreased lymphocytes are commonly associated with stress responses in poultry [20]. Accordingly, the H:L ratio is widely used as a physiological indicator of stress in birds [21], with higher values typically observed under stressful conditions [22]. Peak egg production typically exceeds 90% at around 42 weeks of age [23], which may increase metabolic and physiological demands and, consequently, elevate stress indicators [2]. The interaction between breed and age further suggested that WL hens exhibited higher H:L ratios during peak-laying, whereas LB hens showed more stable hematological profiles across ages. These findings indicate that breed-specific physiological responses may contribute to differences in stress resilience and productive performance in laying hens. In addition, environmental and management factors such as nutrition, housing conditions, and environmental stressors may further influence immune responses and health status in poultry [24].

### mRNA expression of *TLR2* gene

This study provides original comparative data demonstrating that both breed and physiological stage significantly modulate hematological traits and *TLR2*-mediated innate immunity in laying hens, revealing patterns not previously reported in the literature. The higher *TLR2* expression in WL compared to LB hens is a novel finding that may reflect differences in genetic selection pressure. Commercial lines like LB have been intensely selected for egg production, potentially at the cost of certain innate immune traits, whereas purebred WL may retain more robust baseline *TLR2* responsiveness. Unlike Tantikositruj *et al.* [11], who evaluated *TLR2* expression in indigenous chickens, the present study focused on commercial and purebred laying hens during different physiological stages, demonstrating that both genetic background and age may contribute to variation in innate immune-related gene expression. *TLR2* is an important pattern-recognition receptor that detects PAMPs and initiates innate immune responses. When animals are infected or tissue barriers are compromised, microbial components can bind to *TLR2* and activate intracellular signaling pathways, including nuclear factor kappa B (NF-κB), which regulates inflammatory responses and stimulates the production of pro-inflammatory mediators such as cyclo-oxygenase-2 (COX-2), inducible nitric oxide synthase (iNOS), and cytokines including interleukin-1β (IL-1β), interleukin-6 (IL-6), and tumor necrosis factor-α (TNF-α) [25]. *TLR2* is an important pattern-recognition receptor involved in the early detection of microbial components and the initiation of innate immune responses [7, 8, 25–27]. In the present study, *TLR2* expression was higher in WL hens than in LB hens and was greater at 42 weeks than at 80 weeks of age, with the highest expression observed in the WL × 42 group. Previous studies have reported that *TLR2* expression varies among chicken breeds and may be influenced by age, genetic background, and physiological status [28–31]. The higher *TLR2* expression observed in younger laying hens may indicate greater innate immune responsiveness during the peak-laying period. Increased *TLR2* expression may also reflect activation of innate immune pathways associated with physiological stress, inflammatory processes, or exposure to subclinical microbial challenges. Notably, hens at 42 weeks of age exhibited higher heterophil percentages and H:L ratios, which are commonly regarded as indicators of physiological stress in poultry. Therefore, the elevated *TLR2* expression observed in the WL × 42 group may represent a combination of increased immune responsiveness and stress-related immune activation. However, because only *TLR2* gene expression was evaluated in the present study, further investigations involving additional immune-related genes and cytokines are required to clarify the underlying molecular mechanisms. The significant positive correlation between the H:L ratio and *TLR2* expression suggests that birds with higher stress-related hematological responses also tended to show increased *TLR2 *expression. However, further studies including additional inflammatory biomarkers are required to clarify the biological mechanisms underlying this association.

## CONCLUSION

In conclusion, this study demonstrated significant effects of breed and age on hematological parameters and *TLR2* gene expression in laying hens. WL hens exhibited higher hemoglobin concentrations and H:L ratios, while LB hens showed elevated lymphocyte levels. Age-related increases in hematocrit, hemoglobin, lymphocyte, monocyte, and WBC counts were observed at 80 weeks, whereas heterophil counts and H:L ratios were higher at 42 weeks, indicating greater physiological stress during peak production. A significant breed × age interaction was evident, with the WL × 42 group displaying the highest H:L ratio and *TLR2* expression. *TLR2* expression was higher in WL than LB hens and at 42 than at 80 weeks, with the strongest expression in the WL × 42 group. A significant positive correlation was observed between the H:L ratio and *TLR2* gene expression (r = 0.784, p < 0.001), linking stress indicators to innate immune activation. These findings provide novel insights into how genetic background and physiological stage modulate hematological traits and innate immunity during the laying cycle.

The results have practical implications for commercial layer management. Monitoring hematological profiles and *TLR2* expression could serve as non-invasive tools to assess stress and immune status, particularly in high-producing flocks. Breed-specific management strategies, such as tailored nutrition or environmental enrichment during peak-laying, may improve welfare and productivity in WL and LB lines. The study’s strengths include the direct comparison of commercial (LB) and traditional (WL) breeds across defined physiological stages under identical management conditions, as well as the integrated evaluation of hematological and molecular immune markers.

Limitations include the relatively small sample size, focus on only two breeds and a single gene (*TLR2*), and the lack of pathogen challenge or production performance data, which may limit generalizability. Future research should validate these findings in larger cohorts, incorporate multi-gene expression profiling and cytokine analysis, evaluate responses to controlled challenges, and assess long-term effects on egg quality and flock health. Integrating these immune biomarkers into routine monitoring could advance precision poultry management.

Overall, this work highlights the importance of considering both genetic background and production stage when evaluating hen health and immunity, contributing to more sustainable and welfare-oriented layer production systems.

## DATA AVAILABILITY

### The supplementary data can be made available from the corresponding author upon request.

## GENERATIVE AI DECLARATION

The authors declare that generative artificial intelligence (AI) tools were used solely to improve language, grammar, and readability during manuscript preparation. All scientific content, data analysis, interpretation of results, and conclusions were developed and verified by the authors. The authors take full responsibility for the accuracy, integrity, and originality of the work presented, and no AI tool was listed as an author.

## AUTHORS’ CONTRIBUTIONS

TR: Collected the data, performed the investigation, validated the results, and drafted the manuscript. PS: Developed the methodology, supervised the study, and contributed to data visualization. MJU: Supervised the study and contributed to data visualization. AB and NK: Curated the data. AK: Conceived and designed the study, acquired funding, administered the project, supervised the research, and critically reviewed and edited the manuscript. All authors have read and approved the final manuscript.
